# First-in-Human Trial of EphA2-Redirected CAR T-Cells in Patients With Recurrent Glioblastoma: A Preliminary Report of Three Cases at the Starting Dose

**DOI:** 10.3389/fonc.2021.694941

**Published:** 2021-06-21

**Authors:** Qingtang Lin, Teer Ba, Jinyuan Ho, Dandan Chen, Ye Cheng, Leiming Wang, Geng Xu, Lixin Xu, Yiqiang Zhou, Yukui Wei, Jianqiang Li, Feng Ling

**Affiliations:** ^1^ Department of Neurosurgery, Xuanwu Hospital, Capital Medical University, Beijing, China; ^2^ CAR-T Research Center, Hebei Senlang Biotechnology Co., Ltd., Shijiazhuang, China; ^3^ Department of Pathology, Xuanwu Hospital, Capital Medical University, Beijing, China

**Keywords:** chimeric antigen receptor (CAR T), EphA2, glioma, clinical trial, immunotherapy

## Abstract

Glioblastoma is the most common primary brain malignancy with limited treatment options. EphA2 is a tumor-associated-antigen overexpressed in glioblastoma. Pre-clinical studies have demonstrated the promise of EphA2-redirected CAR T-cells against glioblastoma. We conduct the first-in-human trial of EphA2-redirected CAR T-cells in patients with EphA2-positive recurrent glioblastoma and report the results of three patients enrolled as the first cohort receiving the starting dosage (1×10^6^ cells/kg). A single infusion of EphA2-redirected CAR T-cells was administrated intravenously, with the lymphodepletion regimen consisting of fludarabine and Cyclophosphamide. In two patients, there was grade 2 cytokine release syndrome accompanied by pulmonary edema, which resolved completely with dexamethasone medication. Except that, there was no other organ toxicity including neurotoxicity. In both the peripheral blood and cerebral-spinal-fluid, we observed the expansion of CAR T-cells which persisted for more than four weeks. In one patient, there was a transit diminishment of the tumor. Among these three patients, one patient reported SD and two patients reported PD, with overall survival ranging from 86 to 181 days. At the tested dose level (1×10^6^ cells/kg), intravenously infusion of EphA2-rediretected CAR T-cells were preliminary tolerable with transient clinical efficacy. Future study with adjusted dose and infusion frequency of CAR T-cells is warranted.

**Trial Registration Numbers:**

NCT 03423992

## Background

Nearly half of primary brain tumor are gliomas. Most glioma will evolve into their most malignant form, glioblastoma (1). Even with the recent advancements in surgery, radiotherapy, and chemotherapy, the prognosis for patients with glioblastoma remains poor, with a median overall survival of 14.6 months (2). Most glioblastoma will relapse after conventional therapies. Upon recurrence, there is no standard treatment and the median overall survival for recurrent glioblastoma patients is around three months (3).

Immunotherapy has emerged as a breakthrough for the treatment of human cancer (4). Especially, the chimeric antigen receptor T cells (CAR T-cells) therapy has changed the paradigm of treatment for patients with hematologic malignancy (5). However, the application of CAR T-cells in solid tumors remains to be explored. Up to date, there have been four clinical studies evaluating 31 glioblastoma patients who have received CAR T-cells immunotherapy targeted against IL13R2, EGFRvIII, and HER-2 respectively (6–9).

Erythropoietin-producing human hepatocellular carcinoma (Eph) receptor was first identified from hepatocellular carcinoma (HCC) cell line in 1987 (10). Eph receptors constitute the largest tyrosine-kinase-receptor family in the human genome and were classified into A(A1-A8) and B(B1-B6) sub-groups. Upon binding to their ligands (ephrin A1-6 and ephrin B1-3), the Eph/ephrin will activate the downstream signaling and participate in the physiology process of cellular mobility, angiogenesis, and development (11). Most tissues including the brain did not express EphA2 except the epithelial cells of the lung. In contrast, various types of tumor cells including glioblastoma up-regulated EphA2 expression, which makes EphA2 an attractive target for immunotherapy (12). Pre-clinical studies have demonstrated the feasibility and efficacy of EphA2-redirected CAR T-cells for the treatment of a variety of tumors including glioma (13).

**Figure 1:**
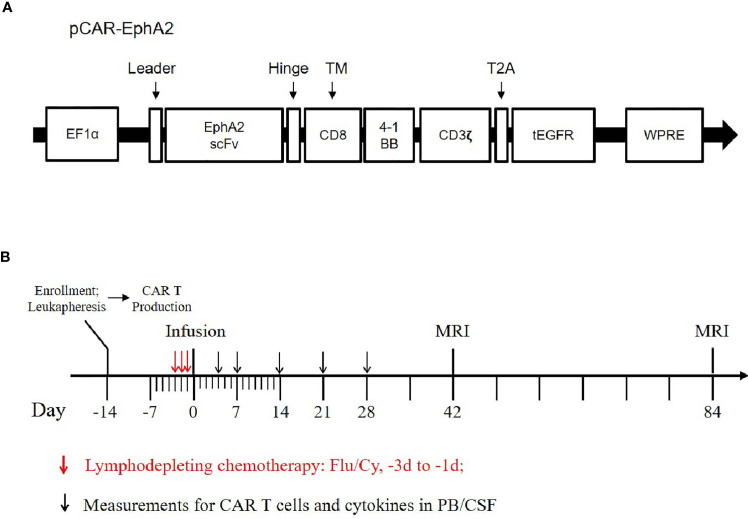
Schema of EphA2-CAR vector and study protocol. **(A)** Sequence diagram of humanized anti-EphA2 CAR vector. EphA2 scFv, humanized anti-EphA2 single-chain fragment variable; tEGFR, truncated epidermal growth factor receptor. **(B)** Study protocol diagram. Flu, Fludarabine; Cy, Cyclophosphamide.

Based on these, we conducted a single-arm, dose-escalation, first-in-human pilot trial of EphA2-redirected CAR T-cells in patients with recurrent EphA2-positive glioblastoma. The primary endpoint of this study is feasibility and safety, secondary endpoint is clinical response and survival. This protocol was approved by the Ethical Committee of Xuanwu Hospital affiliated with Capital Medical University and registered at ClinicalTrials.gov (NCT 03423992). All patients provided written informed consent. The schema of our CAR T-vector was shown in **Figure 1A**, with the *in vitro* functional validation provided in the **Supplementary Figure 1**. A single-dose of EphA2-redirected CAR T-cells was administrated intravenously, with the prior lymphodepletion regimen consisting of Fludarabine (25 mg/m2, d -3 to d -1) and Cyclophosphamide (250 mg/m2, d -2 to d -1) (**Figure 1B**). Toxic effects were monitored according to the National Cancer Institute Common Terminology Criteria for Adverse Events (version 4.X.) (14). Cytokine release syndrome (CRS) was evaluated by the consensus of ASTCT (15). Clinical response was assessed by iRANO criteria (16). Here, we report the preliminary results of the first cohort of three patients receiving the starting dose (1×10^6^ cells/kg).

## Case Presentations

### Patient 1

**Figure 2:**
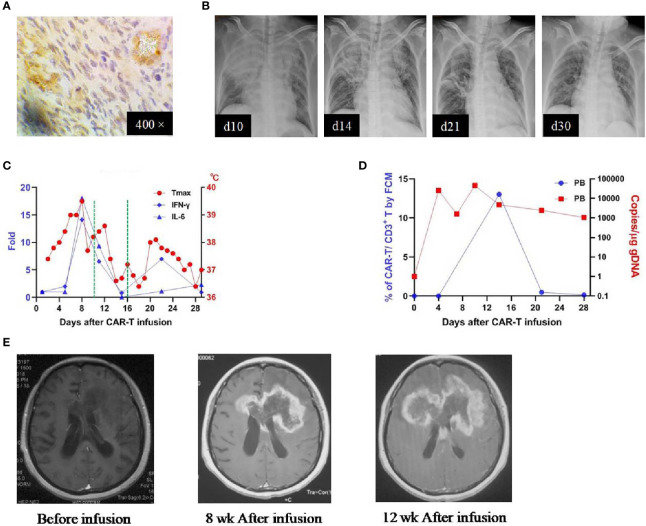
IHC staining of the tumor and clinical response in patient 1. **(A)** Anti-EphA2 IHC staining of tumor tissue before enrollment. **(B)** Thoracic X-ray performed on days 10, 14, 21, after the infusion of CAR T-cells, showing the development and resolution of pulmonary edema in the right superior and middle lobes. **(C)** Dynamic changes of critical cytokines in the peripheral blood along with temperature, after the infusion of CAR T-cells. The dotted green line on the x-axis indicating the time window for the use of dexamethasone. **(D)** Expansion of CAR T-cells in the peripheral blood by flow cytometry (left y-axis) and qPCR (right y-axis). **(E)** Contrast-enhanced MRI performed before and after (8 and 12 weeks) the infusion of CAR T-cells.

Patient 1 was a 45-year-old woman diagnosed with a left frontal glioblastoma in Feb. 2017. She received surgical resection, followed by radiotherapy with concomitant and adjuvant temozolomide chemotherapy. Four months after the surgery, MRI revealed recurrence of the glioblastoma involving left frontal and corpus callosum, for which she was referred to our clinical trial. On evaluating her tumor sample before enrollment, the immunohistochemistry (IHC) study demonstrated an EphA2-positive glioblastoma (**Figure 2A**). She received the CAR T-cells infusion on June 22, 2018 (profile of her CAR T-cells was described in **Supplementary Table 1**). Four days after the infusion, she developed a high fever (lasted for 7 days) and hypotension (on day 7). Simultaneously, there were elevations of several cytokines in the plasma: IL-6 (peaked on day 7 at 478.23 mg/mL, ~18-fold over baseline), IFN-γ (peaked on day 7 at 913.44 pg/mL, ~14-fold over baseline), TNF-alpha (peaked on day 10 at 32.66pg/mL, ~6-fold over baseline), and other cytokines (MCP-1, IL-8, IL-10, IL-18). Thoracic X-ray performed on day 10 revealed pulmonary edema in her right middle and lower lobes (**Figure 2B**). A grade 2 CRS was classified according to the guidelines (15). Beginning on day 10, she was given dexamethasone (5mg for 3 days, then titrated to 2mg for 3 days). She became afebrile on day 11 and the cytokines returned to baseline within 3 weeks (**Figure 2C**). Re-checked thoracic X-rays showed a gradual resolution of her pulmonary edema, which recovered completely on day 30 (**Figure 2B**). There was no other organ toxicity including neuro-toxicity observed. The complete profile of her laboratory investigations was attached in the **Supplementary Table 2**. In the peripheral blood (PB), the CAR T-cells underwent a transient expansion that peaked on day 13 by flow cytometry (FCM) and day 10 by qPCR and persisted at least for 28 days (**Figure 2D**). Re-checked MRI performed 8 weeks after the infusion demonstrated an enlargement of the contrast-enhanced lesion surrounding the tumor in the geneu of corpus callosum; MRI performed 12 weeks after the infusion confirmed the further progression of the tumor (**Figure 2E**). This patient was reported as progressive disease (PD) with an overall survival time of 181 days.

### Patient 2

**Figure 3:**
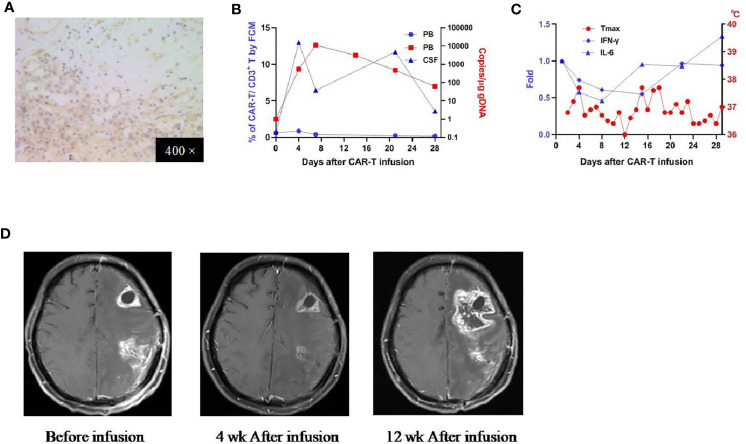
IHC staining of the tumor and clinical response in patient 2. **(A)** Anti-EphA2 IHC staining of tumor tissue before enrollment. **(B)** Expansion of CAR T-cells in the peripheral blood by flow cytometry (left y-axis) and qPCR (right y-axis). **(C)** Dynamic changes of critical cytokines in the peripheral blood along with temperature, after the infusion of CAR T-cells. **(D)** Contrast-enhanced brain MRI performed before and 4 weeks,12 weeks after the infusion of CAR T-cells.

Patient 2 was a 38-year-oldy man who received his first craniotomy in April 2017 for a left frontotemporal glioblastoma, which was followed by radiotherapy with concomitant and adjuvant temozolomide chemotherapy. He underwent another two craniotomies (on Dec. 2017 and June 2018 respectively) because of tumor recurrence, which were later supplemented with traditional Chinese medicine. He was referred to our clinical trial due to the tumor residue. IHC studies revealed an EphA2 positive glioblastoma (**Figure 3A**). He received the infusion on August 14, 2018 (CAR T-cells profile in **Supplementary Table 1**). There was no obvious CRS or other organ cytotoxicity observed on this patient. In peripheral blood, CAR T-cells were detected on day 4, peaked on day 9, and persisted for more than 28 days (by qPCR; not detectable by FCM) (**Figure 3B**). In the serum, there was no elevation of the measured cytokines and most laboratory findings were within the normal range (**Figure 3C** and **Supplementary Table 3**). After the infusion, brain MRI performed at four weeks revealed diminishment of the two enhancing lesions located in the left frontal and parietal lobes respectively; MRI performed at 12 weeks demonstrated the progression of the frontal lesion and the sustained diminishment of the parietal lesion (**Figure 3D**). He reported as SD at 4 weeks, with an OS of 164 days.

### Patient 3

**Figure 4:**
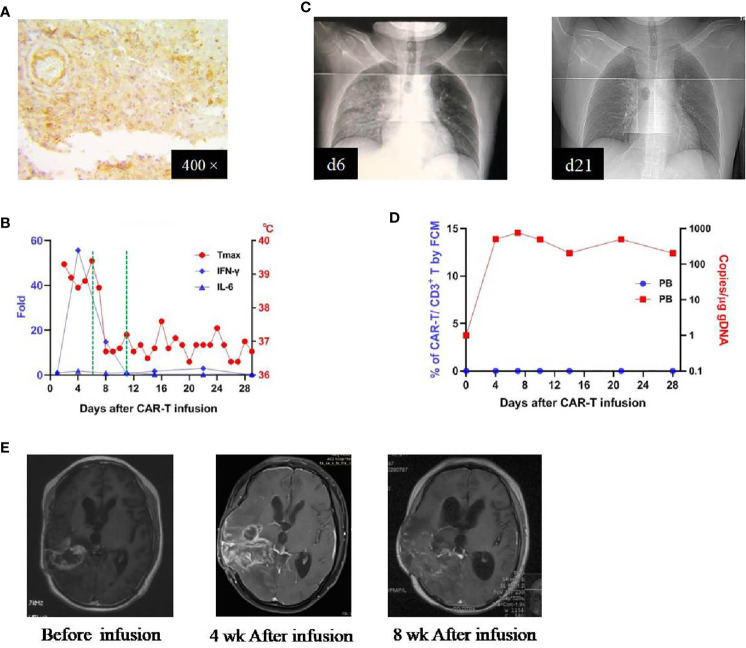
IHC staining of the tumor and clinical response in patient 3. **(A)** Anti-EphA2 IHC staining of tumor tissue before enrollment. **(B)** Dynamic changes of critical cytokines in the peripheral blood along with temperature, after the infusion of CAR T-cells. The dotted green line on the x-axis indicating the time window for the use of dexamethasone. **(C)** Thoracic X-ray performed on days 6 and 21 after the infusion of CAR T-cells, showing the development and resolution of pulmonary edema in the right middle and inferior lobes. **(D)** Expansion of CAR T-cells in the peripheral blood by flow cytometry (left y-axis) and qPCR (right y-axis). **(E)** Contrast-enhanced MRI performed before and after (4 and 8 weeks) the infusion of CAR T-cells.

Patient 3 was a 30-year-old man who received his first craniotomy on Feb. 2017 for a right-sided frontotemporal glioblastoma, which was followed by a standard treatment regimen. He underwent his second craniotomy in November 2017 due to tumor recurrence and received chemotherapy thereafter. He participated in our clinical trial because of tumor residue (**Figure 4A**) and was administrated CAR T-cells on August 28, 2018 (CAR T-cells profile in **Supplementary Table 1**). This patient developed a high fever on day 1 (7 hours after the infusion), which lasted for 6 days. Meanwhile, there were elevations of several cytokines, with the IFN-γ as the most dramatic (peaked on day 4, ~56-fold over baseline) (**Figure 4B**). Pneumonia edema was observed on day 6 and resolved completely on day 21 with the administration of dexamethasone (5 mg, given on days 6-11) (**Figure 4C**). After the use of dexamethasone, he became afebrile on day 7 and the plasma cytokines returned to normal within 3 days. In peripheral blood, CAR T-cells were detected on day 4, peaked on day 7, and lasted more than 28 days (by qPCR; not detectable by FCM) (**Figure 4D**). There were no other toxicities and most laboratory findings were within the normal range (**Supplementary Table 4**). MRI study performed 4 weeks after infusion revealed enlargement of the tumor, which was not reduced obviously 4 weeks later (**Figure 4E**). This patient was reported as PD with an OS of 86 days.

## Discussion and Conclusion

We conducted a first-in-human trial of EphA2-redirected CAR T-cells administrated intravenously in patients with recurrent EphA2-positive glioblastoma and reported the results of the first three patients enrolled in the starting dose. Two patients experienced graded 2 CRS (patients 1 and 3). One patient achieved SD and two patients reported PD, with the OS ranging from 86 to 181 days.

Previous studies of CAR T-cells against glioblastoma have administrated the CAR T-cells initially through intra-cranial (local-regional) (6, 7), and then later by intra-venous (8, 9). The intra-cranial approach needs the implantation of an infusion catheter, which is an invasive procedure and can be associated with certain complications (i.e., infection and bleeding). The intra-venous approach is relatively less invasive, and results of previous studies have shown that intravenously infused CAR T-cells could cross the blood-brain-barrier and reach the targeted glioma tissue (8). Based on these, we preferentially administrated our CAR-T-cells *via* intravenous route.

One of the major safety concerns for CAR T-cells immunotherapy has largely resided in the expression of the targeted antigens. Among two of these three patients, pulmonary edema was observed, accompanied by the occurrence of high fever and elevations of the relevant cytokines. This lung adverse effect was not associated with hemodynamic change. With the use of dexamethasone, this pulmonary edema was resolved completely within 3 weeks, and most of the cytokines returned to normal. Except this, there was no other organ toxicity. As mentioned previously, EphA2 is a tumor-associated-antigen, which under the physiology condition is also expressed by lung tissues. Pre-clinical study of EphA2-redirected CAR T-cells against established lung metastasis animal model has demonstrated that the intravenously infused CAR T-cells could traffic into the lung tissue (17). Therefore, it is possible that the observed pulmonary edema in these patients could due to the “on-target, off-tumor” effect. However, we could not completely rule out the possibility of “off-target, off-tumor” lung organ cytotoxicity.

In the peripheral blood, there was a transient expansion of EphA2-redirected CAR T-cells in all three patients, with the peak values ranging from day 7 to day 10. CAR T-cells could persist for more than 4 weeks. The qPCR seemed to be more sensitive than the flow cytometry in detecting the expansion of CAR T-cells. In previous four CAR T-cells studies of glioblastoma with different targeted antigens (6–9), one reported similar transient expansion in the peripheral blood of intravenous-infused EGFRvIII-directed CAR T-cells (8). Two studies targeted IL13R2 did not observe peripheral expansion of CAR T-cells, which was reasonable, based on their intra-cranial infusion approach (6, 7). Underlying the observed proliferation of EphA2-redirected CAR-T-cells in the peripheral blood, one possible reason is the application of the lymphodepletion regimen, while previous studies did not. There have been several reports showing the role of lymphodepletion in boosting the proliferation of CAR T cells (18). Another potential reason is the reactivity of EphA2-redirected CAR T-cells with the EphA2-expressing epithelial cells of the lung, which subsequently stimulate its proliferation. Among these three patients, the amplification of CAR T-cells in patient 1 was most substantial; correspondingly, the pulmonary edema was most severe. Due to the unavailability of tumor tissue, we were unable to measure the proliferation of CAR T-cells in the brain of these three patients. However, in patient 2, we were able to obtain the CSF and observe a robust proliferation of CAR T-cells in the CSF, indicating the ability of EphA2-redirected CAR T-cells to cross the blood-brain-barrier and proliferate in the host tumor microenvironment. Prior study has shown that the expansion of CAR T-cells in the CSF was associated with the proliferation of CAR T-cells in glioma tissus (7). Therefore, to some extent, the measurement of CAR T-cells in the CSF may potentially serve as a surrogate for the evaluation of the CAR T-cells in the targeted glioma tissue.

The current standard for the measurement of glioma’s response is based on the MRI study. However, the application of this standard in the setting of immunotherapy seems to be complex, because of the so-called “pseudo-progression” resulted from the immune response. We use the iRANO criteria in assessing the tumor response in these three patients. Before enrollment into our clinical trial, all three patients have been heavily pre-treated and underwent multiple recurrences, indicating an unfavorable prognosis. With a single infusion, one patient was reported SD at initial follow-up. In patient 2, the administration of a single dose CAR T-cells resulted in the diminishment of the tumor. Of note, this “transit response” sustained less than one month, which was parallel to the persistence of the CAR T cells. To further improve the clinical efficacy, multiple infusions with an adjusted dose of CAR T-cells would be considered in our future study.

In conclusion, intravenous infusion of EphA2-redirected CAR T-cells at the tested dose (1×10^6^ cells/kg) in these three patients was tolerable with transit efficacy. Future study with adjusted dose and infusion frequency is warranted to further investigate the safety and efficacy of EphA2-redirected CAR T-cells for the treatment of glioblastoma.

## Data Availability Statement

The original contributions presented in the study are included in the article/**Supplementary Material**. Further inquiries can be directed to the corresponding authors.

## Ethics Statement

The studies involving human participants were reviewed and approved by Ethical Committee of Xuanwu Hospital affiliated with Capital Medical University. The patients/participants provided their written informed consent to participate in this study. Written informed consent was obtained from the individual(s) for the publication of any potentially identifiable images or data included in this article.

## Author Contributions

QL wrote the original manuscript draft. TB and JH performed the study. DC provided technical support and participated in the collection and analysis of the data. YC, GX, LX, YZ, and YW took care of the patients. LW contributed and reviewed the pathology study. JL, FL, and QL directed this research. All authors contributed to the article and approved the submitted version.

## Conflict of Interest

JH and JL were employed by Hebei Senlang Biotechnology Co., Ltd.

The remaining authors declare that the research was conducted in the absence of any commercial or financial relationships that could be construed as a potential conflict of interest.
